# Supplementary material for: Patterns of Intron Gain and Loss in Fungi
Source: PLoS Biol. 2004 Nov 30;2(12):e422. doi: 10.1371/journal.pbio.0020422 (PMC532390; doi:10.1371/journal.pbio.0020422)
Supplement: Table S1 — Also available at http://genes.mit.edu/NielsenEtAl/. (4.3 MB ZIP). [file pbio.0020422.st001.zip › NielsenEtAl/html/182.html]

AN7489.1.NCU00056.1.MG10202.1.FG07226.1


```
 CLUSTAL W (1.82) Multiple Sequence Alignments - Introns Inserted


Sequence 1: NCU00056.1	435 aa
Sequence 2: FG07226.1	427 aa
Sequence 3: MG10202.1	447 aa
Sequence 4: AN7489.1	430 aa
Alignment Length: 469 aa
Number Identitical Residues: 202 aa
Alignment Score (without introns) 10604


MG10202.1 	------MSLRRVVVTGIG-AVT---PLGVG~AQRSWQRLIAGASGITSVTHLEPLDRWQE
NCU00056.1	--------MRRVVVTGLG-AIT---PLAVG~IRPTWRRLLDSHSGIVSVADLEPQDKWKE
FG07226.1 	--------MRRVVVTGLG-AIT---PLGVG~VKRTWTRLLSGECGIVSVADLEPQTRWKE
AN7489.1  	MILQLSPSISNVSTSRKGRSITGADASKQG1VRRTWKRLLDGHCGIVNIRHRD--ARYAD
          	   . : :: .* .:  * ::*.:..   *  : :* **: . .**..: . :   :: :

MG10202.1 	LPSTVAGLVPTSAAADANANAAKSHGGAGDVDGGKASGLWRPSDWLDAT~DQRRMSRFAQ
NCU00056.1	LTSTVGGLVPTPARNKQNVDEQHT---------------WKAADWLEPS~DQRRMSLFAQ
FG07226.1 	LTSTVSGLVPS-------GDGEGQ---------------WRASDWLSAN~EQRRMSKFTQ
AN7489.1  	LPCEIAAVVPSG------ARAEGR---------------WTASEWLSKD0DERKMARFAQ
          	*.. :..:**:                            * .::**.   ::*:*: *:*

MG10202.1 	YALASADMALEDARWMPETPHDLEATGVCLGSGIGNLEEIYNTALAHHK-~---------
NCU00056.1	YSVAATEMALQDAGWKPTRQEDLEATGVCMGSGIGNLDDFYNTSVTYDKE0GYKRVSPLF
FG07226.1 	YAIAASDMALKDAGWEPKSEEQLEATGVCLGSGIGNLDEIYDTSLVHHKD0GYKKVSPLF
AN7489.1  	YAMAAAEEALEDAEWKPKAEDQLEATGVCLGSGIGNFDEIYDAVVAYDTK~GYKKLSPLF
          	*::*::: **:** * *   .:*******:******::::*:: :.:... . .  :.  

MG10202.1 	--H0ILINLAAGHIAMHHGFRGPNHAATTACTTGAHSIGDAARFIAFGDADVMVAGGSES
NCU00056.1	VPK~ILINLGAGHIAMRYGFRGPNHSATTACTTGAHSIGDASRFIAFGDADVMVAGGSES
FG07226.1 	VPK~ILINMAAGHIAMKYGFQGPNHAATTACTTGAHSIGDASRFISMGDADIMVAGGSES
AN7489.1  	VPK~LLINLGAGHISMKYGFMGPNHSATTACTTGAHSIGDAARFIACGDADVMLAGGAES
          	 .: :***:.****:*::** ****:***************:***: ****:*:***:**

MG10202.1 	CIHPLTFAGFARSRSLSTAFNDNPTASCRPFDESRDGFVVAEGAAVVVLEELE~HALRRG
NCU00056.1	CIHPLTFAGFGRSRSLSTAYNHDPTSACRPFDKDRAGFVVSEGAAVLILEELE~HAKRRG
FG07226.1 	CIHPLTFAGFGRARSLSTAYNNNPTASCRPFDADRNGFVVSEGAAVLILEELE~HAKARG
AN7489.1  	CIHPLAIGGFARARSLATAYNDRPEKSSRPFDADRDGFVVGE----------E1HAKARG
          	*****::.**.*:***:**:*. *  :.**** .* ****.*          * **  **

MG10202.1 	ARILAELRGYGCSGDAHHMTAPREDGSGALAAMKKALKHAGVRPAEVDYINAHATGTRSG
NCU00056.1	ARILAELTGYGCSGDAHHMTAPRDDGSGAFLSMKRALKNAGVKPGDVDYINAHATGTAVG
FG07226.1 	ARIYAEIKGYGCSGDAHHMTAPREDGRGAFLAMKKALKNAGVKPSQVDYINAHATATNVG
AN7489.1  	ARIYAEVKGYGCSGDAHHMTAPKANGGGALLAMKKALKNACLEPSAVDYINAHATSTVIG
          	*** **: **************: :* **: :**:***:* :.*. *********.*  *

MG10202.1 	DAAEALAIRSLMLGEEGLEREDQVTVSSTKGSTGHLLGAAGAVEAVFTALAVAE0NRVPP
NCU00056.1	DAAEAIAIRSLMMGEEGVDDESRITVSSTKGAIGHLLGAAGAIESVFSILAIAE0GAIPP
FG07226.1 	DVAETSAIRRLMLGEEGHEKESDITVSSTKGAVGHLLGAAGAIEALFSVLAIHQ0RVVPA
AN7489.1  	DAAENAAIKALLLGPGGKQKAADVNVSSTKGAIGHLLGGAGAVEALFTVLAIHE0NVMPP
          	*.**  **: *::*  * :    :.******: *****.***:*::*: **: :   :*.

MG10202.1 	TKNLFNPGVGPNFNFVANQTQERTVNVAVSNSFGFGGTNASLVFSKLERGMQGSI
NCU00056.1	TLNLTSPNVGVGFNFVPVQAQQKEVKVAMSNSFGFGGTNASLVFSKFE-------
FG07226.1 	TLNLQKPDVGAAFNFVPNAAQERNVGVAVSNSFGFGGTNSSLVFSKY--------
AN7489.1  	TINLDRLNDGFDFNYAPNEAQERRIDLALTNSFGFGGTNSSLCFSKHGL------
          	* **   . *  **:..  :*:: : :*::*********:** ***
```
